# Parents’ Participation in School Health Examinations for Their Adolescent Children in Finland

**DOI:** 10.1177/10598405211058841

**Published:** 2021-12-13

**Authors:** Hanne M. Kivimäki, Timo P. Ståhl, Katja M. Joronen, Arja H. Rimpelä

**Affiliations:** 1 The Department of Public Health and Welfare, 3837 Finnish Institute for Health and Welfare, Helsinki, Finland; 2 8058 Department of Nursing Science University of Turku, Turku, Finland; 3 Faculty of Social Sciences, Unit of Health Sciences, 7840 Tampere University, Tampere, Finland; 4 Department of Adolescent Psychiatry, 7840 Tampere University Hospital, Tampere, Finland

**Keywords:** health/wellness, school-based clinics, middle/junior/high school, quantitative research

## Abstract

Engaging parents in school health examinations can promote adolescents’ well-being. We examined parents’ participation in universal school health examinations in Finland reported by adolescents in school surveys (14 to 16-year-olds, *N* = 58,232). Further we studied variation between service providers and schools, and student and school-level factors in participation. National data were analyzed using multilevel logistic regression models. Less than half of the adolescents reported parents’ participation. The variation between service providers and schools was large. Non-participation was associated with mother's low education, students’ immigrant background, daily health complaints, heavy drinking, and discussion difficulties with parents. Boys and those who did not live with both mother and father had a higher risk for parents’ non-participation. Adolescents with a long-term illness or being bullied reported participation more often. Inviting parents and the school health nurse resource were not associated with participation. Our results raise the question of barriers to participation in health examinations.

Well-functioning school health services have a great opportunity to meet most adolescents and promote their health and well-being (Baltag et al., 2015; Leroy et al., 2017; Mason-Jones et al., 2012). Cooperation with families and parents is recommended in the context of school health services to guarantee the quality of services (National Association of School Nurses, 2016; World Health Organization, 2014). Communication with parents can help get a better view of children's well-being and socio-demographic background, which has a major effect on children's lives (Ristikari et al., 2018), and strengthen the effectiveness of health promotion activities (Busch et al., 2013; Ford et al., 2011; Kuntsche & Kuntsche, 2016). Parents’ engagement in health services is important especially during adolescence when developmental changes and possible risk behavior may create the need for support and guidance (Patton et al., 2016).

One way for parents’ involvement is their participation in students’ regular health examinations, which are common in European school health services (Michaud et al., 2021; Rimpelä et al., 2013). In Finland, municipalities are service providers for universal school health services, and each municipality organizes these services for one or several schools in their area. Services are free of charge, and all 7 to 16-year-old students (1st–9th graders) are met in annual health examinations. By law (Government Decree, 2011), three of them are comprehensive health examinations, namely those at the 1st, 5th, and 8th grades. Parents are invited to these to discuss the well-being of the whole family, get the parent's assessment of their child's well-being and obtain information and advice. Comprehensive health examinations include visits to both the school health nurse and the school doctor, and students have an opportunity for confidential conversation without parents. (Hakulinen-Viitanen et al., 2012; Rimpelä et al., 2013).

Most Finnish service providers report organizing all three comprehensive health examinations (TEAviisari, 2021a). Nevertheless, a study among school health nurses has shown that parents’ participation is much rarer in the 8th-grade health examination for 14 to 15-year-old adolescents than in younger students’ examinations. The parents’ participation rate is also known to vary greatly between service providers and schools. (Hietanen-Peltola et al., 2019). Furthermore, earlier studies have shown a regional variation in school health services’ resources (Wiss et al., 2017) and adolescents’ perceived access to services (Kivimäki et al., 2019).

It is known that organizational, financial, social or cultural factors have an influence on the utilization of health services (Gulliford et al., 2002). Little is known of parents’ participation in health examinations in school health services, but research has revealed barriers and facilitators to parents’ involvement in school health services or school-based health promotion programs. First, the organization of school health services matters. The service provider's time constraints, competing demands, or system requirements may hinder parents’ participation (Helitzer et al., 2011; Spencer et al., 2018). Likewise, parents’ lack of information about the services, an inconvenient appointment time, or late notification of the appointment time may be barriers (Silván et al., 2014).

Second, the parents’ or families’ situation may prevent their participation. Limited resources—for example, difficulties with the schedule, time constraints, family demands, or the high cost of care may be barriers to participation (Garcia-Dominic et al., 2010; Silván et al., 2014). Parents’ negative attitudes towards health examinations or school health services overall (Silván et al., 2014) and perceived low value of services (Garcia-Dominic et al., 2010) may also decrease participation.

Third, adolescents’ health and well-being may have an influence. The participation may be lower if parents get information about their adolescent's health from a source other than the school health services or they think that their child and the family are doing well (Silván et al., 2014). On the other hand, among younger children, a child's worse health status has increased the risk for non-participation in health examinations (Gibb et al., 2019). Likewise, parents do not necessarily participate if they trust the adolescent's maturity to handle their own issues and want to give them privacy (Silván et al., 2014). Parents may participate less often if the adolescent is reluctant to their inclusion or parents suppose that their presence might make the discussion more difficult or that the adolescent might be embarrassed (Silván et al., 2014). On the other hand, efforts to ensure confidentiality might have an effect on participation and discussions of sensitive matters—parents’ involvement might be difficult to promote if they do not approve of the possible risk behavior or are not aware of it (Helitzer et al., 2011).

Finally, several socio-demographic factors are associated with parents’ participation. Fathers and older parents seem to participate less in comprehensive health examinations in school health services, and single or unemployed parents participate slightly more often (Halme et al., 2013). On the other hand, the younger children's participation rate in the universal health examination or similar has been found to be lower, if either mother (Gibb et al., 2019) or both parents are young (Søndergaard et al., 2008) or if the child has a single parent (Søndergaard et al., 2008). Likewise, participation seems to be lower if the children are from socioeconomically deprived areas (Gibb et al., 2019; Wood et al., 2012), parents’ have a low educational level, have a decreasing household income, or are outside of the labor market (Søndergaard et al., 2008). Cultural differences or language may be barriers to parents’ involvement, too (National Association of School Nurses, 2016). Younger children seem to participate less in child health examinations if they have a refugee or immigrant background (Moller et al., 2016), but this association may vary by children's age (Søndergaard et al., 2008).

The purpose of this study was to examine whether parents participate in health examinations of their adolescent children and whether there is variation between schools or service providers in parents’ participation rate. We also examine whether school health service organization or students’ socio-demographic background, health, and well-being explain parents’ non-participation. The factors studied were i) school health service organization: the school health nurse resource and the parents’ invitation to comprehensive health examinations, ii) students’ socio-demographic background: students’ gender, parents’ unemployment, mother's education, student's residence (living with mother and father), immigrant background, and iii) students’ health and well-being: daily health complaints, long-term illness, heavy drinking, weekly bullying, and discussion difficulties with parents.

## Method

### Participants

Two nationwide data sources were used: the student-level data were obtained from the School Health Promotion study (SHP) and the school-level data from the Benchmarking System of Health Promotion Capacity Building (BSHPCB) data collection for comprehensive schools.

The SHP study is a nationwide survey that has monitored Finnish adolescents’ health and well-being since 1996. The study is conducted by the Finnish Institute for Health and Welfare every second year, and the participation is free of charge for schools and municipals. All Finnish lower secondary schools with students in the 8th and 9th grade were invited to the study. Data for this study was collected in April–May 2017 as an anonymous and voluntary online classroom survey. In total, 63% of all Finnish 8th and 9th graders (*N* = 73,680) participated in the study. (Finnish Institute for Health and Welfare, 2020; Finnish Institute for Health and Welfare, 2021).

The SHP study data collection in 2017 was approved by the ethical committee of the Finnish Institute for Health and Welfare (THL/1704/6.02.0 1/2016). The students were informed of the aim and content of the survey. Students had an opportunity to decline participation, and the survey was anonymous. In addition, the parents and guardians were informed, and they could opt out of their child's participation by informing the teacher. (Finnish Institute for Health and Welfare, 2020; Finnish Institute for Health and Welfare, 2021).

The BSHPCB is a nationwide Finnish benchmarking tool for local governments and schools. The data collection is organized by the Finnish Institute for Health and Welfare and the Finnish National Agency for Education. The purpose is to manage, plan, and evaluate health promotion activities and resources. The data used were collected by principals from comprehensive schools with grades 1–9 or 7–9 in collaboration with the school welfare team. A total of 91% of comprehensive schools (*N* = 735) participated in October–November 2017. (TEAviisari, 2021b; Wiss et al., 2018).

The SHP study and the BSHPCB data sets were linked for this study using a unique school number given to each school. Additionally, the information about the service provider was obtained from the BSHPCB data collection from 142 municipal health centers (97%) in 2018 (TEAviisari, 2021a). To minimize possible bias in the school-level results, we excluded schools with fewer than 10 students in the 8th and 9th grades (*n* = 50 schools, 227 students) and likewise special education schools (*n* = 28 schools, 480 students) due to the students’ special needs for support, and schools with missing information about the service provider in the BSHPCB data (*N* = 10 schools, 889 students).

### Measurement

#### Outcome Variable

Students were asked when they had had a health examination and if their parents participated in it. Parents’ participation was measured by the question, “Was either of your parents with you at the health examination?”, with five possible answers (“Yes, in 7th grade; Yes, in 8th grade; Yes, in 9th grade; No; Don’t know”). Respondents could choose one option. The variable “Parents’ participation in health examination” was dichotomized as “No” (“No”) and “Yes” (Yes, in the 7th/8th/9th grade). The outcome variable was “Parents’ non-participation in health examination” (participation/non-participation).

We included in our analyses those students who reported their grade, those who had a health examination by a school health nurse or a school doctor in grades 7–9, and those who reported parents’ participation (yes/no) in the health examination in grades 7–9. According to school health nurses, the 8th-grade comprehensive health examination may have been organized during the summer holidays or even during the previous school year (Hietanen-Peltola et al., 2019). Altogether, 21% of the students (*N* = 15,112) were excluded from the data for missing or invalid values for these questions. Most of them did not report attending a health examination in grades 7–9 with a school health nurse or school doctor (*N* = 8,911) or parent's participation in a health examination (*N* = 4,152) or did not know if their parents participated in it (*N* = 6,498).

#### School-Level Variables

School-related variables from the BSHPCB data were the school health nurse resource and the parents’ invitation to the comprehensive health examinations. These described the organization of the school health services.

The school health nurse resource was originally measured by the nurse-to-student ratio and categorized into three groups: (1) 0–600 students/school health nurse; (2) over 600 students/school health nurse; and (3) not reported. The national recommendation is no more than 600 students per one full-time school health nurse (Ministry of Social Affairs and Health, 2004), which is 20 days or 140.25 h of work per month.

Schools were also asked if parents were invited to the 8th-grade comprehensive health examinations. A three-scaled parents’ invitation (yes/no/not reported) was used as the covariate.

#### Student-Level Variables

Variables from the SHP study described students’ socio-demographic background, health, and well-being. Most of them were dichotomized to make the scales convergent.

Four variables represented the students’ socio-demographic background. Mother's education was categorized into a two-level variable (high/low). Parent's unemployment (no/yes) refers to whether at least one parent had been unemployed or laid-off during the past 12 months. Living with a mother and father refers to whether the student lived with them both in one home (yes/no). Student's immigrant background was categorized into three categories, according to the students’ and parents’ birth countries: (1) Finnish background (at least one parent born in Finland); (2) Immigrant background, born in Finland (student born in Finland and the only parent or both parents outside of Finland); (3) Immigrant background, born outside of Finland (both student and the only parent or both parents born outside of Finland). In addition to these, the student's gender (girl/boy) was used as a covariate.

Students’ health and well-being were described with five dichotomized (yes/no) variables. “At least two daily health complaints” included the following health complaints: neck or shoulder pain; lower back pain; stomach ache; trouble falling asleep or waking up during the night; headache; tiredness or dizziness; stuffy nose or runny nose; dry or sore throat; cough; dry or watery eyes. Long-term illness refers to chronic illness or a health problem diagnosed by a physician. Health behavior was described by heavy drinking at least once a month, meaning alcohol being consumed at least once a month to get heavily drunk. Weekly bullying described perceived bullying at school during that semester. Discussion difficulties with parents meant that the student could hardly ever talk about personal things with parents.

### Data Analysis

The frequencies of the covariates were first examined. The proportion of students who reported parents’ participation was studied by service providers and schools. Bivariate associations between covariates and the outcome variable were studied with chi-square test. Multilevel logistic regression model was used to evaluate school- and student-level covariates as fixed effects on the parents’ participation reported by students. Because the data were hierarchical, information about service providers and schools were included in the analysis to adjust for random effects (Heck et al., 2012). The association of school- and student-level covariates with parents’ non-participation was studied with multivariate models.

The strength of each covariate's association with parents’ non-participation was described in an odds ratio (OR) at a 95% confidence level. The analyses were conducted using SPSS version 27. The level of significance was set at 0.05.

## Results

### Characteristics of the Sample

The final data consisted of 58,232 students (50.0% of all Finnish 8th and 9th graders) from 653 schools (80.4% of all comprehensive schools for grades 1–9 or 7–9); 45.7% of them were boys and 54.3% girls.

Distributions of variables describing the organization of school health services are presented in Table 1, and variables describing students’ socio-demographic background, health, and well-being are in Table 2. Most schools reported 600 students or fewer per one full-time school health nurse and parents’ invitation to the comprehensive health examination. The total number of students includes those who did not report their gender. Since the frequencies of covariates were mostly at the same level for boys and girls, genders were combined in the analysis.

**Table 1.** Distributions of Schools (*N*  =  653) and Students (*N*  =  58,232) According to the Organization of School Health Services.

School-level variable		Schools	Students		
		(*N* = 653) *n* (%)	Boys (*N* = 26,507) *n* (%)	Girls (*N* = 31,460) *n* (%)	Total (*N* = 58,232) *n* (%)
School health nurse resource					
	600 students or less per school health nurse	458 (70.1)	18,472 (69.7)	22,102 (70.3)	40,748 (70.0)
	Over 600 students per school health nurse	103 (15.8)	4,200 (15.8)	4,872 (15.5)	9,114 (15.7)
	Not reported	92 (14.1)	3,835 (14.5)	4,486 (14.3)	8,370 (14.4)
Parents’ invitation to the comprehensive health examination					
	Yes	545 (83.5)	22,139 (83.5)	26,214 (83.3)	48,558 (83.4)
	No	44 (6.7)	1,639 (6.2)	2,073 (6.6)	3,739 (6.4)
	Not reported	64 (9.8)	2,729 (10.3)	3,173 (10.1)	5,935 (10.2)

Note. Those (*n*  =  265) who did not report their gender were included in the total.

**Table 2.** Distributions of Boys and Girls According to Socio-Demographic and Health and Well-Being Variables (*N*  =  58,232).

Student-level variable		Boys (*N* = 26,507) *n (%)*	Girls (*N* = 31,460) *n (%)*	Total (*N* = 58,232) *n (%)*
Socio-demographic background				
Mother's education				
	High	16,128 (65.1)	19,685 (65.9)	35,959 (65.5)
	Low	8,644 (34.9)	10,187 (34.1)	18,921 (34.5)
Parents’ unemployment				
	No	18,467 (71.0)	21,070 (67.7)	39,708 (69.2)
	Yes	7,541 (29.0)	10,073 (32.3)	17,698 (30.8)
Living with mother and father				
	Yes	18,737 (71.4)	21,408 (68.4)	40,319 (69.8)
	No	7,492 (28.6)	9,898 (31.6)	17,474 (30.2)
Immigrant background				
	Finnish background	24,818 (95.4)	29,833 (95.6)	54,890 (95.5)
	Immigrant background and born in Finland	422 (1.6)	615 (2.0)	1,043 (1.8)
	Immigrant background and born outside of Finland	782 (3.0)	767 (2.5)	1,563 (2.7)
Health and well-being				
At least two daily health complaints				
	No	24,039 (93.5)	24,891 (80.8)	49,111 (86.5)
	Yes	1,663 (6.5)	5,928 (19.2)	7,650 (13.5)
Long-term illness				
	No	21,256 (81.2)	23,827 (76.3)	45,287 (78.6)
	Yes	4,909 (18.8)	7,395 (23.7)	12,363 (21.4)
Heavy drinking at least once a month				
	No	23,901 (91.1)	28,702 (91.8)	52,842 (91.5)
	Yes	2,331 (8.9)	2,551 (8.2)	4,904 (8.5)
Weekly bullying				
	No	25,044 (95.0)	30,081 (96.0)	55,364 (95.5)
	Yes	1,309 (5.0)	1,259 (4.0)	2,590 (4.5)
Discussion difficulties with parents				
	No	24,796 (95.5)	28,443 (91.3)	53,466 (93.2)
	Yes	1,164 (4.5)	2,705 (8.7)	3,900 (6.8)

Note. Those (*n*  =  265) who did not report their gender were included in the total.

### Parents’ Participation in Health Examinations

Less than half (44.1%) of the students (*N* = 58,232) reported that parents had participated in their health examination: 14.8% in the 7th grade, 27.7% in the 8th grade, and 1.7% in the 9th grade.

The proportion of students who reported parents’ participation ranged between service providers (*N* = 141) from 1.4% to 83.4% (mean 44.7%, median 45.7%) and between schools (*N* = 653) from 0.0 to 91.6% (mean 42.8%, median 43.2%).

Parents’ participation was less common (Table 3) if the school had not reported the school health nurse resource and parents were not invited to the comprehensive health examination. Likewise, students reported less participation by parents if they were boys, the mother had low education, they did not live with both mother and father, they did not have a Finnish background, and parents had been unemployed during the last year. Parents’ participation was also less prevalent among those who had at least two daily health complaints, did not have a long-term illness, drank alcohol heavily at least once a month, were not bullied weekly, or had discussion difficulties with parents. Based on cross-tabulation, the reference category for long-term illness and weekly bullying was changed for further analysis. All studied variables had a statistically significant association with parents’ participation.

**Table 3.** Percentage of Parents who did not Participate in the School Health Examination According to the Organization of School Health Services, Student's Socio-Demographic, and Health and Well-Being Variables (*N*  =  57,366–58,232).

Variable	Parents’ non-participation in the health examination		
	%	*n*	*p* Value
School-level variables			
School health nurse resource			
600 students or less per school health nurse	54.9	40,748	.000
Over 600 students per school health nurse	55.8	9,114	
Not reported	61.0	8,370	
Parents’ invitation to the comprehensive health examination			
Yes	55.2	48,558	.000
No	59.6	3,739	
Not reported	59.5	5,935	
Student-level variables			
Socio-demographic background			
Gender			
Girl	54.1	31,460	.000
Boy	58.0	26,507	
Mother's education			
High	54.2	35,959	.000
Low	58.5	18,921	
Parents’ unemployment			
No	55.4	39,708	.002
Yes	56.8	17,698	
Living with mother and father			
Yes	54.9	40,319	.000
No	58.2	17,474	
Immigrant background			
Finnish background	55.4	54,890	.000
Immigrant background and born in Finland	64.9	1,043	
Immigrant background and born outside of Finland	66.9	1,563	
Health and well-being			
At least two daily health complaints			
No	55.5	49,111	.000
Yes	58.5	7,650	
Long-term illness			
No	56.5	45,287	.000
Yes	53.8	12,363	
Heavy drinking at least once a month			
No	55.4	52,842	.000
Yes	61.4	4,904	
Weekly bullying			
No	56.0	55,364	.002
Yes	52.9	2,590	
Discussion difficulties with parents			
No	55.2	53,466	.000
Yes	64.9	3,900	

*Note.* Level of significance for chi-square-test < .05.

Further analysis showed the associations between the organization of school health services and parents’ non-participation (Table 4). Both bivariate and multivariate models showed that school health nurse resources under the national recommendations (600 students per one nurse) did not have a statistically significant association with parents’ non-participation. Similarly, if the school health nurse resource was not reported, no association with parents’ participation was found in the multivariate model. Students from schools that did not invite parents to the comprehensive health examination or did not report this information reported participation by parents was less likely, but these associations were not statistically significant in the multivariate model.

**Table 4.** Associations Between the Organization of School Health Services, Students’ Socio-Demographic Background, Health, and Well-Being With Parents’ Non-participation in the School Health Examination (*N*  =  58,232).

Variables			Bivariate models		Multivariate model	
			(*N* = 54,880–58,232)		(*N* = 51,453)	
			OR [95% CI]	*p* Value	OR [95% CI]	*p* Value
School-level variables						
School health nurse resource						
	600 students or less per school health nurse		Ref.		Ref.	
	Over 600 students per school health nurse		1.04 [0.99–1.09]	.091	1.03 [0.86–1.24]	.755
	Not reported		1.29 [1.22–1.35]	.000	1.07 [0.81–1.42]	.644
Parents’ invitation to the comprehensive health examination						
	Yes		Ref.		Ref.	
	No		1.20 [1.12–1.28]	.000	0.99 [0.76–1.29]	.935
	Not reported		1.20 [1.13–1.26]	.000	1.00 [0.72–1.39]	.994
Student-level variables						
Socio-demographic background						
Gender						
	Girl		Ref.		Ref.	
	Boy		1.17 [1.14–1.21]	.000	1.26 [1.21–1.31]	.000
Mother's education						
	High		Ref.		Ref.	
	Low		1.19 [1.15–1.24]	.000	1.22 [1.16–1.27]	.000
Parents’ unemployment						
	No		Ref.		Ref.	
	Yes		1.06 [1.02–1.10]	.002	0.98 [0.94–1.02]	.382
Living with mother and father						
	Yes		Ref.		Ref.	
	No		1.14 [1.10–1.18]	.000	1.18 [1.13–1.23]	.000
Immigrant background						
	Finnish background		Ref.		Ref.	
	Immigrant background and born in Finland		1.49 [1.31–1.69]	.000	1.47 [1.25–1.71]	.000
	Immigrant background and born outside of Finland		1.63 [1.46–1.81]	.000	1.64 [1.44–1.88]	.000
Health and well-being						
At least two daily health complaints						
	No		Ref.		Ref.	
	Yes		1.13 [1.08–1.19]	.000	1.13 [1.06–1.20]	.000
Long-term illness						
	Yes		Ref.		Ref.	
	No		1.11 [1.07–1.16]	.000	1.13 [1.08–1.18]	.000
Heavy drinking at least once a month						
	No		Ref.		Ref.	
	Yes		1.28 [1.20–1.36]	.000	1.28 [1.19–1.37]	.000
Weekly bullying						
	Yes		Ref.		Ref.	
	No		1.14 [1.05–1.23]	.002	1.23 [1.11–1.35]	.000
Discussion difficulties with parents						
	No		Ref.		Ref.	
	Yes		1.50 [1.40–1.61]	.000	1.53 [1.41–1.66]	.000

*Note.* Odds ratios (OR) and their 95% confidence intervals (95% CI). Multivariate logistic regression model adjusted for service provider and school. Level of significance *p* < .05.

All selected student-level covariates had a statistically significant association with parents’ non-participation in the bivariate model. The multivariate model revealed almost similar associations when the service provider and school were adjusted. Most of the socio-demographic covariates were associated with parents’ participation. Boys were more likely to report parents’ non-participation than girls, as did those whose mother had a low education compared to those whose mother had a high education. Parents’ unemployment had a statistically significant association with parents’ non-participation only in the bivariate model. Students who did not live with both mother and father reported lower participation by parents than others. Students with an immigrant background, both born in Finland and outside of Finland, reported less parent participation than those with Finnish background.

All covariates related to students’ health and well-being were associated with parents’ non-participation. Having at least two daily health complaints, drinking alcohol heavily at least once a month, and having discussion difficulties with parents were associated with parents’ non-participation. Students with no long-term illness or no weekly bullying reported non-participation more often. This means that students who had a long-term illness or were bullied weekly reported parents’ participation more often than those without it.

## Discussion

Less than half (44%) of the adolescent students reported parents’ participation in the health examination. Parents’ participation was less likely if the mother had low education, the student did not live with both mother and father, or the student had an immigrant background. Furthermore, participation was less likely if the student was a boy, had at least two daily health complaints, got heavily drunk at least monthly, or had discussion difficulties with parents. Students who were bullied or had a long-term illness reported parents’ participation more often. The parents’ participation varied widely between service providers and schools, but the school health nurse resource was not related with the participation.

Our result of parents’ low participation rate is consistent with Finnish school health nurses’ assessment of parents’ participation in the adolescents’ comprehensive health examination (Hietanen-Peltola et al., 2019). Furthermore, the parents’ participation rate varied greatly between schools and service providers. Similar results have been found in previous Finnish studies (Kivimäki et al., 2019; Wiss et al., 2017). The school health nurse resource or the parents’ invitation to health examinations had no statistically significant association with parents’ participation. On the other hand, there was no information about how the resources were allocated or in which format or language the invitations were sent. However, in Finland most schools use electronic communication systems to interact with parents and school health nurses often use these for direct messages to parents. In addition, it was not known if parents got the invitation in time or if they had the possibility to choose a suitable time for the appointment, both of which have an influence on participation (Silván et al., 2014). Our results reflect a variation in school health services that needs further investigation.

According to our results, mother's low education was associated with parents’ non-participation. In line with our study, previous research (Gibb et al., 2019; Søndergaard et al., 2008; Wood et al., 2012) has shown that the odds for non-participation are higher in families with lower socioeconomic backgrounds. Likewise, our finding that an immigrant background was associated with non-participation is consistent with the existing literature (Moller et al., 2016). These results raise the concern that parents from these groups might face barriers to participation. World Health Organization (2014) has recommended that accessibility, equity, and acceptability need be considered when engaging with parents in school health services. These services should be accessible to all parents despite their socio-demographic background or possible language barriers (Schyve, 2007).

A previous study showed that a child's lower health status increased the risk for non-participation in the health examination (Gibb et al., 2019). According to our study, parents participated more often if their child had a long-term illness or was bullied. Still, students with several daily health complaints more often reported parents’ non-participation. Student's chronic illness or bullying might be more familiar to parents than self-reported health complaints, and this creates the need for cooperation with health services. Regular health examinations can give a chance to discuss of all these matters.

One of the tasks of health counseling in the Finnish school health services is to support parenting and the interaction between parents and children (Government Decree, 2011). The association of discussion difficulties with parents’ non-participation might reflect a previous finding that adolescent's reluctance to parents’ participation or embarrassment might prevent it (Silván et al., 2014). Likewise, previous research has implied that efforts toward confidentiality might create challenges to parents’ participation (Helitzer et al., 2011). In our study, heavy drinking was associated with non-participation. Nevertheless, health examinations can give family members a chance to discuss health and well-being and also risk behavior, but still offer the adolescent the important possibility for a confidential conversation with school health services personnel (Patton et al., 2016; Irwin, 2018).

Our findings highlight that parents’ non-participation in the health examination was associated not only with the socio-demographic background but also with the student's health, well-being, and gender. Furthermore, we found great differences between service providers (1.4–83.4%) and schools (0.0–91.6%) in the parents’ participation rate. These results raise the question of the parents’ possibilities to participate in the health examinations. More knowledge is needed about strategies and practices of parent engagement in school health services. It would be useful to extend our findings by examining how parents are invited, reached, and informed about the health examination's purpose and their role in it.

### Implications for School Nursing

A couple of recommendations based on our study findings can be given for school nursing practice in efforts to reach parents and increase the possibilities to access services.

First, parents’ participation in the health examination can be supported in many ways. According to our study, less than half of the students reported parents’ participation, and non-participation was associated, for example, with socio-demographic background. Previous studies have shown that both the service provider and parents’ time constraints (Garcia-Dominic et al., 2010; Helitzer et al., 2011; Spencer et al., 2018; Silván et al., 2014) may prevent participation. Flexible scheduling of health examinations and an innovative use of digital services (for instance, parents’ participation via remote connection) might promote their involvement. Since parents’ health literacy and digital literacy level may vary, the information needs to be given in an easily understandable and accessible format. Immigrant background was associated with non-participation, and therefore using an interpreter should also be considered.

Second, there was a wide variation between both service providers and schools in the parents’ participation rate. Systematic and planned approaches can promote participation (Centers for Disease Control & Prevention, 2012; Helitzer et al., 2011). Legislation or national guidelines determine the organization of school health services, but local, shared practices for contacting parents would be beneficial, too. Attention needs to be given to how meetings are organized, and appointments scheduled.

Finally, the purpose of cooperation and participation in health examinations should be emphasized to parents. Since school health services are usually located on the school premises and adolescents do not need parents to access the services, their participation might be difficult to promote (Helitzer et al., 2011; Rimpelä et al., 2013). According to a previous study, the lack of information about services might prevent participation (Silván et al., 2014). The school health services might be familiar to parents, but the potential in supporting families during adolescence may not be clear to them. The importance and benefits of these services for both parents and their children should be highlighted.

### Strengths and Limitations

This research is based on nationwide studies that represent most Finnish lower secondary schools and their students. The School Health Promotion study is anonymous and voluntary, and the students had the possibility to answer in five languages. Students, who were absent from school, for example because of travel, illness or truancy, are not represented (Finnish Institute for Health and Welfare, 2020).

However, there are three potential limitations of this study. First, the special education schools were not selected for our analysis due to the students’ special need for support. This means that students with the most severe inabilities or learning difficulties are not represented in our study. Other students with difficulties in reading or understanding the text had the possibility to answer a shorter and simplified questionnaire (Finnish Institute for Health and Welfare, 2021) that did not include questions about health examinations.

A second potential limitation concerns the question of health examinations. Students were asked about the health examination and their parents’ participation, but it is not known if they had a comprehensive health examination where their parents were invited. Nevertheless, most service providers reported organizing 8th grade comprehensive health examinations and inviting parents to them. In addition, most students reported participating in the health examination in the 8th or 9th grade. Analyses were made separately for 8th and 9th graders in case the larger proportion of older students had had a comprehensive health examination and therefore would report more parents’ participation, but no great differences were found.

Third, several students were excluded from the analyses because of missing or invalid answers. For example, 8.8% of the students did not know if their parents participated in a health examination. Since the latest health examination may have been organized over a year before responding to the SHP study, students may have had difficulties in recalling if their parents participated.

Despite these limitations, this research describes students’ experiences in school health examinations targeted to both adolescents and their parents. The results can be utilized in organizing school health services for families from different backgrounds or with challenges for well-being.

## Conclusions

Less than half of the students reported parents’ participation in the school health examination, and the variation between schools or service providers was large. Both adolescent well-being and several socio-demographic factors were associated with non-participation. Our results highlight barriers to participation in health examinations.
